# ChatGPT does make up scientific references: should it be currently renamed CheatGPT?

**DOI:** 10.1007/s00228-024-03758-7

**Published:** 2024-09-10

**Authors:** Gerd Mikus

**Affiliations:** Heidelberg University Hospital – Medical Clinic (Krehl-Klinik), Internal Medicine IX - Department of Clinical Pharmacology and Pharmacoepidemiology, Im Neuenheimer Feld 410, 69120 Heidelberg, Germany

In the latest issue of the journal, the performance of ChatGPT in clinical rule-guided dose interventions in hospitalized patients with renal impairment was investigated and found to be poor [1]. I am working on yohimbine pharmacokinetics and the involvement of CYP2D6 on its clearance [2] where an update of my current knowledge was necessary. In addition to the usual literature search using PubMed and other online databases, the idea was to ask ChatGPT to maybe gain some more information which is available but not easy to find. For this purpose, I used the free ChatGPT Version 1.2024.170 on a Mac, which is GPT-4o.

First question: “*What is the relationship between yohimbine and CYP2D6?*”

Answer: “Yohimbine is an alkaloid derived from the bark of the Yohimbe tree, often used for its purported effects on erectile dysfunction and weight loss. Its relationship with the enzyme CYP2D6 is significant in terms of its metabolism and pharmacokinetics.”

1. Metabolism: Yohimbine is metabolized by …
2. Genetic Variability: CYP2D6 exhibits genetic polymorphism …
3. Drug Interactions: Co-administration of yohimbine with …
4. Clinical Implications: Understanding an individual's CYP2D6 genotype …

In summary, CYP2D6 is a key enzyme in the metabolism of yohimbine, and variations in CYP2D6 activity due to genetic polymorphism or drug interactions can significantly affect the pharmacokinetics and clinical outcomes of yohimbine therapy.

Next questions were as follows:

“*Are there specific scientific publications available regarding these 4 topics?*”

“*Is there more published on metabolism of yohimbine by CYP2D6?*”

ChatGPT came up with 5 references.

“*Can you give these references with a link and a PubMed ID?*”

Answer: Certainly! Here are the references with their respective PubMed IDs and links to access them:

1. “Characterization of the Human Cytochrome P450 Enzymes Involved in the In Vitro Metabolism of Yohimbine”
  Authors: Venkatakrishnan K, von Moltke LL, Greenblatt DJ
  Journal: British Journal of Clinical Pharmacology (2002)
  PubMed ID: 12392574
  Link: [Characterization of the Human Cytochrome P450 Enzymes Involved in the In Vitro Metabolism of Yohimbine](https://pubmed.ncbi.nlm.nih.gov/12392574/)
2. “In vitro inhibition of human liver microsomal cytochrome P4502D6 by yohimbine and its derivatives”
  Authors: Delaforge M, Jaouen M, Bouille G
  Journal: European Journal of Drug Metabolism and Pharmacokinetics (1995)
  PubMed ID: 8694884
  Link: [In vitro inhibition of human liver microsomal cytochrome P4502D6 by yohimbine and its derivatives](https://pubmed.ncbi.nlm.nih.gov/8694884/)
3. “The role of cytochrome P450 enzymes in the metabolism of yohimbine in humans”
  Authors: Williams LL, Harris RZ, Ferrell TL, Greenblatt DJ
  Journal: Journal of Pharmacology and Experimental Therapeutics (1996)
  PubMed ID: 8646566
  Link: [The role of cytochrome P450 enzymes in the metabolism of yohimbine in humans](https://pubmed.ncbi.nlm.nih.gov/8646566/)
4. “Metabolism of yohimbine by human liver microsomes and recombinant human cytochrome P450 enzymes”
  Authors: Obach RS, Reed-Hagen AE
  Journal: Drug Metabolism and Disposition (2002)
  PubMed ID: 12167556
  Link: [Metabolism of yohimbine by human liver microsomes and recombinant human cytochrome P450 enzymes](https://pubmed.ncbi.nlm.nih.gov/12167556/)
5. “Comparative study of yohimbine metabolism by human, monkey, and dog liver microsomes”
  Authors: Polasek TM, Miners JO
  Journal: Drug Metabolism and Disposition (2006)
  PubMed ID: 16467192
  Link: [Comparative study of yohimbine metabolism by human, monkey, and dog liver microsomes](https://pubmed.ncbi.nlm.nih.gov/16467192/)

Since I know many of the authors, I was surprised that they should have been working on the same topic without me knowing about it. The authors were not made up by ChatGPT; they really exist and publish in the area of drug metabolism. However, all publication titles do not exist whereas the journals are the target journals for this type of research. All provided PubMed IDs exist; however, these are references which have different authors, different topics, and different journals. The conclusion is (at least currently) as follows: do not use ChatGPT for this purpose and definitely do not rely on any references provided without verifying. Instead, more sophisticated AI-powered tools like scite.ai or perplexity.ai might be useful.

## Data Availability

No datasets were generated or analysed during the current study.
